# Risk Factors for the Development of Neurological Deficits in Metastatic Spinal Disease: An International, Multicenter Delphi Study

**DOI:** 10.1177/21925682231222424

**Published:** 2025-01-12

**Authors:** Eline H. Huele, Roxanne Gal, Wietse S.C. Eppinga, Helena M. Verkooijen, John E. O’Toole, Ilya Laufer, Daniel M. Sciubba, Cordula Netzer, Wouter Foppen, Arjun Sahgal, Michael G. Fehlings, Sheng-fu L. Lo, Charles G. Fisher, Laurence D. Rhines, Jeremy J. Reynolds, Aron Lazary, Alessandro Gasbarrini, Nicolas Dea, Michael H. Weber, Jorrit Jan Verlaan

**Affiliations:** 1 Division of Imaging and Oncology, 8124 University Medical Center Utrecht, Utrecht University, Utrecht, The Netherlands; 2 Department of Radiotherapy, Division of Imaging and Oncology, 8124 University Medical Center Utrecht, Utrecht University, Utrecht, The Netherlands; 3 Department of Neurosurgery, 2468 Rush University Medical Center, Chicago, IL, USA; 4 Division of Spinal Neurosurgery, Department of Neurosurgery, NYU Langone Health, New York, NY, USA; 5 Department of Neurosurgery, 232890 Donald and Barbara Zucker School of Medicine at Hofstra/Northwell, Great Neck, NY, USA; 6 Department of Spine Surgery, 30262 University Hospital Basel, Basel, Switzerland; 7 Department of Radiology and Nuclear Medicine, Division of Imaging and Oncology, 8124 University Medical Center Utrecht, Utrecht University, Utrecht, The Netherlands; 8 Department of Radiation Oncology, Odette Cancer Centre, 71545 Sunnybrook Health Sciences Centre, University of Toronto, Toronto, ON, Canada; 9 Division of Neurosurgery and Spine Program, University of Toronto and Toronto Western Hospital, Toronto, Canada; 10 Division of Spine Surgery, Vancouver General Hospital and University of British Columbia, Vancouver, BC, Canada; 11 Department of Neurosurgery, Division of Surgery, 4002 The University of Texas MD Anderson Cancer Center, Houston, TX, USA; 12 Spinal Division, 6397 Oxford University Hospital NHS Trust, Oxford, UK; 13 National Center for Spinal Disorders and Buda Health Center, Budapest, Hungary; 14 Department of Spine Surgery, 18509 IRCCS Istituto Ortopedico Rizzoli, Bologna, Italy; 15 Department of Surgery, McGill University, Montreal, QC, Canada

**Keywords:** metastatic spinal disease, neurological deficits, risk factors, delphi

## Abstract

**Study Design:**

Delphi study

**Objective:**

The objective of this study was to identify risk factors associated with the development and/or progression of neurological deficits in patients with metastatic spinal disease.

**Methods:**

A three-round Delphi study was conducted between January-May 2023 including AO Spine members, comprising mainly neurosurgeons and orthopedic surgeons. In round 1, participants listed radiological factors, patient characteristics, tumor characteristics, previous cancer-related treatment factors and additional factors. In round 2, participants ranked the factors on importance per category and selected a top 9 from all factors. Kendall’s W coefficient of concordance was calculated as a measure of consensus. In the final round, participants provided feedback on the rankings resulting from round 2. Lastly, the highest-ranking factors were more clearly defined and operationalized by an expert panel.

**Results:**

Over two hundred physicians and researchers participated in each round. The factors listed in the first round were collapsed into 12 radiological factors, 14 patient characteristics, 6 tumor characteristics and 12 previous cancer-related treatment factors. High agreement was found in round 3 on the top-half lists in each category and the overall top 9, originating from round 2. Kendall’s W indicated strong agreement between the participants. ‘Epidural spinal cord compression’, ‘aggressive tumor behavior’ and ‘mechanical instability’ were deemed most influential for the development of neurological deficits.

**Conclusion:**

This study provides factors that may be related to the development and/or progression of neurological deficits in patients with metastatic spinal disease. This list can serve as a basis for future directions in prognostication research.

## Introduction

Sixteen percent of patients with solid tumors develop metastatic spinal disease and one third of patients with metastatic epidural spinal cord compression develop neurological deficits.^1^ The first symptoms in patients with metastatic spinal disease are often non-specific, which, if left unrecognized, can lead to unremitting pain and eventually neurological symptoms.^2^ Neurological symptoms often require emergency surgery to deter or delay progression of, or reverse, neurological deficits.^3^ Delayed surgical treatment of patients with spinal metastases has been demonstrated to be associated with more frequent and dense neurological deficits, which have a large and negative impact on clinical and patient-reported outcomes including quality of life and survival.^2,4,5^ Neurological deficits are often irreversible but may be preventable by timely recognition of patients at risk.

Most patients who develop symptomatic spinal metastases have a known malignancy.^6^ Increased awareness of alarming symptoms indicative for the development of neurological deficits, may lead to faster recognition, expedited referral to an appropriate center for definitive care and more effective treatment, which may in turn improve outcomes.^2^ However, recognizing patients at increased risk of neurological deficits, preferably before the onset of neurological deficits, is challenging. The mechanisms involved in the development of neurological deficits in metastatic spinal disease may be more complex than simple mechanical compression of the spinal cord (or cauda equina) and may involve complex interactions, for example, the morphological configuration and location of the epidural mass, local or regional mechanical instability, spinal cord perfusion, effects from previous local irradiation, and potentially other (unknown) factors, including genetic susceptibility to neurological dysfunction.

To the best of our knowledge, there is a paucity of literature on factors that are predictive for the development of neurological deficits in metastatic spinal disease, as well as on factors that may interact and/or have a synergistic effect. The primary objective of this Delphi study is to use expert knowledge and opinion to identify factors perceived (or thought to) be associated with the development and/or progression of neurological deficits in patients with metastatic spinal disease.^7^ As the available literature is scarce, expert consensus can help to identify factors that may be related to neurological deficits from spinal metastases. The results from this study can be used as a starting point to build a predictive model for the development of neurological deficits in patients with metastatic spinal disease.

## Methods

### Study Design

The Delphi method is an established iterative method to obtain consensus from a group of experts.^8-10^ We performed a three-round Delphi study to generate a list of risk factors that may be important for the development and/or progression of neurological deficits in metastatic spinal disease, followed by consensus on the importance of the risk factors and identification of interactions between often compounding risk factors. Lastly, we defined the risk factors within an expert panel.

### Participants

The questionnaires were distributed to active members of AO Spine, an international group including dedicated spine surgeons and (radiation) oncologists. AO Spine is a global academic community for innovative education and research for the purposes of improving spine care and patient outcomes. In every round, AO Spine members were invited to participate by e-mail which included a link to the questionnaire. As the survey was filled out anonymously, it was not possible to identify participants who completed the previous round(s). Therefore, all participants also received an e-mail invitation for the second and third round, regardless of response to the first and/or second round. During every round, the invitees received one e-mail reminder asking to complete the questionnaire. After the final round, an expert panel, consisting of members from the AO Spine Knowledge Forum Tumor Instability Workgroup, defined the highest-ranking risk factors.

### Procedure

In round 1, an open-ended questionnaire was sent to the participants asking them to list radiological factors, patient characteristics, tumor characteristics, previous cancer-related treatment factors and additional factors they believed to be associated, directly or indirectly, with the development and/or progression of neurological deficits in metastatic spinal disease. The questionnaire was sent on January 06, 2023 and closed January 22, 2023.

In round 2, the participants were asked to rank the radiological factors, patient characteristics, tumor characteristics, and previous cancer-related treatment factors, that resulted from the first round, in order of importance for the development of neurological deficits in patients with metastatic spinal disease. Then the participants were asked to construct a top 9 ranking of importance from all factors. Lastly, the participants were asked to indicate possible interactions between two or three often compounding factors for the development of neurological deficits in patients with metastatic spinal disease. The risk factors were listed in alphabetical order on a single page to minimize bias. The questionnaire was open from March 02, 2023 until March 20, 2023.

In round 3, the top 6 radiological factors, the top 7 patient characteristics, the top 3 tumor characteristics, the top 6 previous cancer-related treatment factors and the top 9 of all risk factors from round 2 were presented to the participants. Participants were asked whether they believed the rankings were correct. They were given the option to rank the factors in their preferred order. This third and final questionnaire could be accessed from April 19, 2023 until May 08, 2023.

### Data Analysis

The surveys were distributed in SurveyMonkey. The open-ended responses from the first round produced lists of radiological factors, patient characteristics, tumor characteristics, pervious cancer-related treatment factors and additional factors. The lead authors, comprising an orthopedic surgeon with a special interest in metastatic spinal disease, a clinical epidemiologist in the field of bone metastases, and a medically trained PhD student, analyzed the data and consolidated all factors. Overlapping factors were combined and terminology was unified, followed by categorization of factors.

After the second round, the ranked lists for each category were analyzed using SPSS version 27.0. Available case analysis was used to handle missing data. For each separate question on the survey, all completed rankings were included in the analysis. A ranking score was calculated for each factor based on their mean rank, which was the sum of all ranking numbers divided by the total number of rankings. A lower mean rank score indicates a higher rating of importance. In addition, the proportion of respondents who ranked the factor in their top-half ranking was determined. Kendall’s coefficient of concordance (W) was calculated to determine the level of agreement between respondents. Scores ranged between 0-1.^11^ A score of .1 indicated very weak agreement, a score of .5 moderate agreement and a score of .7 strong agreement.^12^

To determine the top 9 of all risk factors, a score per factor was computed by (1) multiplying 10 - the ranking number (i.e.*,* a number 1 ranking equals 9 points and a number 9 ranking equals 1 point) by the times being ranked on that position, and (2) summing the results into one score. Next, the score was divided by the highest possible score, which was 249 (ie, number of respondents in round 2) * 9 = 2241, to have a score between 0 and 1. For example, if a factor was 9 times ranked as number 1 and 7 times ranked as number 3, the score of this factor was (9*9 + 7*7)/2241 = .06.

After the third round, the data were analyzed by repeating the steps after the second round to determine final rankings per category and overall top 9 based on the mean rank and the top-half ranking, and to determine consensus based on Kendall’s coefficient of concordance.

### Operationalization of Risk Factors

The factors resulting from round 3 were discussed within an expert panel, consisting of members from the AO Spine Knowledge Forum Tumor Instability Workgroup, during three online meetings. The aim of this expert meeting was to establish definitions and classifications for the factors. In addition, the factors were categorized in one of two groups: risk factors unanimously accepted by experts and supported by literature and risk factors suggested by experts, currently not supported by literature. The expert panel agreed that for the first category of risk factors there is a good understanding of the mechanisms that lead to the development of neurological deficits. Risk factors suggested by experts, currently not supported by literature are risk factors that warrant more research to understand how they contribute or relate to neurological deficits.

## Results

### Characteristics of Participants

The first questionnaire was completed by 211 participants, the second questionnaire by 249 participants and the third and final questionnaire was filled out by 302 participants. The majority of participants were from Europe and Asia, were male, orthopedic surgeons or neurosurgeons and had spine oncology as practice focus. Demographics and expertise of the participants are presented in Table 1.

**Table 1.** Baseline Characteristics of the Participants.

	Round 1		Round 2		Round 3	
	N	%	N	%	N	%
Number of respondents	211		249		302	
Continent						
Africa	19	9.0	28	11.2	28	9.3
Asia	58	27.5	73	29.3	87	28.8
Australia	1	0.5	0	0	1	0.3
Europe	67	31.8	96	38.6	107	35.4
North America	27	12.8	23	9.2	32	10.6
South America	39	18.5	29	11.6	47	15.6
Sex						
Male	192	91.0	227	91.2	283	93.7
Female	17	8.1	21	8.4	17	5.6
Non-binary	1	0.5	0	0	1	0.3
Unknown	1	0.5	1	0.4	1	0.3
Age (in years)						
25-34	24	11.4	48	19.3	50	16.6
35-44	98	46.4	98	39.4	116	38.4
45-54	52	24.6	63	25.3	85	28.1
55-64	27	12.8	27	10.8	38	12.6
65+	10	4.7	13	5.2	13	4.3
Duration of practicing spine surgery (in years)						
<5	41	19.4	67	26.9	77	25.5
5-10	62	29.4	63	25.3	76	25.2
11-15	43	20.4	43	17.3	51	16.9
16-20	22	10.4	29	11.6	40	13.2
>20	43	20.4	47	18.9	58	19.2
Specialty						
Orthopedic surgeon	122	57.8	145	58.2	173	57.3
Neurosurgeon	84	39.8	94	37.8	117	38.7
Radiation oncologist	0	0	1	0.4	1	0.3
Other	5	2.4	9	3.6	11	3.6
Practice focus*						
Degenerative, yes	185	87.6	210	84.3	263	87.1
Deformities, yes	107	50.7	118	47.4	139	46.0
Trauma/Spinal cord injury, yes	165	78.2	181	72.7	226	74.8
Tumor, yes	171	81.0	194	77.9	222	73.5
Practice setting						
Academic/University affiliated	103	48.8	142	57.0	158	52.3
Private practice	39	18.5	31	12.4	50	16.6
Public hospital/Government/military hospital	64	30.3	70	28.1	88	29.1
Other	5	2.4	6	2.4	6	2.0
Number of spine surgeries per year						
<50	21	10.0	33	13.3	42	13.9
51-100	56	26.5	58	23.3	71	23.5
101-150	37	17.5	34	13.7	52	17.2
151-200	26	12.3	47	18.9	50	16.6
201-250	23	10.9	25	10.0	29	9.6
>250	45	21.3	46	18.5	56	18.5
Unknown	3	1.4	6	2.4	2	0.7
Respondents participated in one of previous rounds						
Yes	NA	NA	116	46.6	168	55.6
No	NA	NA	133	53.4	134	44.4

*The sum of responses can be > 100 as more than one answer was possible.

Abbreviations: NA, not applicable.

### Results of Round 1

A total of 703 radiological factors, 615 patient characteristics, 551 tumor characteristics, 387 previous cancer-related treatment factors and 265 additional factors were suggested. After deduplication, 291, 182, 116, 127 and 131 factors, respectively, were categorized by the lead authors into 12 radiological factors, 14 patient characteristics, 6 tumor characteristics and 12 previous cancer-related treatment factors (Table 2). The factors identified as additional factors in the Delphi study were categorized into these four categories.

**Table 2.** Risk Factors Identified in Round 1.

Radiological Factors	Patient Characteristics	Tumor Characteristics	Previous Cancer-Related Treatment Factors
Deformity (kyphosis, malalignment, etc.)	ASA classification ≥3	Aggressive tumor behavior	Absence of multidisciplinary team
Epidural spinal cord compression (ESCC)	BMI <18.5 or >30.0	Hypervascular tumor	Deviation from treatment plan
Inappropriate imaging (eg, radiographs)	Elderly patients	Involvement of ≥2 columns	Exhausted radiotherapy options
Midthoracic location	Frailty	Osteodestructive tumor	Leakage of cement in spinal canal
Lytic lesion	Immunosuppression	Radioresistant tumor	No/inappropriate administration of corticosteroids
Mechanical instability	Low socio-economic status	Widespread metastases	Persisting mechanical instability post-surgery
Poor bone quality	Malnutrition		Poor response to systemic treatment
Posterolateral involvement	Missing alarm symptoms/red flags		Prolonged hypotension perioperatively
Diffuse metastases	Neuropathic pain		Refractory to prior surgery
Tumor compromising vascular supply spinal cord	Poor access to care		Surgeon with little expertise
Spinal cord abnormalities	Smoking/alcohol abuse		Systemic treatment affecting perfusion of spinal cord
Vertebral body collapse	Unable to care for self		Toxicity-induced discontinuation of previous treatment
	Underestimating the urgency of symptoms		
	Young age at diagnosis of primary tumor		

Abbreviations: ASA, American Society of Anesthesiologists; BMI, Body Mass Index.

### Results of Round 2

The second round resulted into rankings of the risk factors per category based on the mean rank (Table 3). The Kendall’s W for radiological factors, patient characteristics, tumor characteristics and previous cancer-related treatment factors were .33, .18, .42 and .22 respectively, indicating weak to moderate agreement.

**Table 3.** Ranked Risk Factors Identified in Round 2.

Rank	Risk Factor	Mean Ranking Score	Top-Half Rank, %	Kendall’s W
	**Radiological factors**			.33
1	Epidural spinal cord compression (ESCC)	2.15	94.7	
2	Mechanical instability	4.27	82.4	
3	Deformity (kyphosis, malalignment, etc)	4.67	77.3	
4	Vertebral body collapse	6.04	64.4	
5	Lytic lesion	6.17	56.3	
6	Midthoracic location	6.26	55.2	
7	Tumor compromising vascular supply spinal cord	6.38	50.7	
8	Posterolateral involvement	7.72	26.9	
9	Diffuse metastases	8.20	25.5	
10	Spinal cord abnormalities	8.34	28.3	
11	Poor bone quality	8.58	16.5	
12	Inappropriate imaging (eg, radiographs)	9.22	21.8	
	**Patient characteristics**			.18
1	ASA classification ≥3	4.92	74.3	
2	Missing alarm symptoms/red flags	5.28	66.1	
3	Elderly patients	5.50	74.0	
4	Frailty	6.11	67.1	
5	Underestimating the urgency of symptoms	6.30	63.6	
6	Immunosuppression	7.18	54.5	
7	BMI <18.5 or >30.0	7.23	54.5	
8	Poor access to care	7.53	47.3	
9	Neuropathic pain	8.04	38.6	
10	Low socio-economic status	8.22	42.3	
11	Malnutrition	8.45	37.6	
12	Unable to care for self	9.27	35.1	
13	Smoking/alcohol abuse	10.29	18.8	
14	Young age at diagnosis of primary tumor	10.67	26.0	
	**Tumor characteristics**			.42
1	Aggressive tumor behavior	1.59	94.6	
2	Involvement of ≥2 columns	2.77	70.8	
3	Osteodestructive tumor	3.34	54.6	
4	Hypervascular tumor	3.88	37.8	
5	Radioresistant tumor	4.65	20.0	
6	Widespread metastases	4.77	22.2	
	**Previous cancer-related treatment factors**			.22
1	Absence of multidisciplinary team	3.65	79.8	
2	Persisting mechanical instability post-surgery	4.75	72.8	
3	Poor response to systemic treatment	5.25	61.3	
4	Deviation from treatment plan	5.27	65.2	
5	Exhausted radiotherapy options	5.35	67.5	
6	Refractory to prior surgery	6.38	50.7	
7	Leakage of cement in spinal canal	7.07	42.4	
8	Prolonged hypotension perioperatively	7.20	34.1	
9	Surgeon with little expertise	7.30	44.4	
10	No/inappropriate administration of corticosteroids	7.87	33.4	
11	Systemic treatment affecting perfusion of spinal cord	8.21	29.8	
12	Toxicity-induced discontinuation of previous treatment	9.69	18.5	

Abbreviations: ASA, American Society of Anesthesiologists; BMI, Body Mass Index.

The top 9 ranking of all risk factors is listed in Table 4. Epidural spinal cord compression (ESCC) was deemed the most important risk factor, with a score of .72. The top 9 ranking consisted of five radiological factors, three tumor characteristics, one patient characteristic and no previous cancer-related treatment factors.

**Table 4.** Top 9 Ranking of all Risk Factors Identified in Round 2.

Rank	Factor	Score 0-1	Category
1	Epidural spinal cord compression (ESCC)	.72	Radiological factor
2	Aggressive tumor behavior	.50	Tumor characteristic
3	Mechanical instability	.40	Radiological factor
4	Deformity (kyphosis, malalignment, etc)	.25	Radiological factor
5	Involvement of ≥2 columns	.22	Tumor characteristic
6	Missing alarm symptoms/red flags	.18	Patient characteristic
7	Tumor compromising vascular supply spinal cord	.17	Radiological factor
8	Osteodestructive tumor	.16	Tumor characteristic
9	Vertebral body collapse	.16	Radiological factor

A total of 806 interactions between risk factors were suggested by the participants, of which 681 interactions comprised three factors and 125 comprised two factors. The interactions were diverse: the majority of interactions were a unique combination of factors. Factors from the overall top 9 ranking were suggested most often in the interactions. Further study to clarify the interactions between factors is ongoing.

### Results of Round 3

The Kendall’s W for radiological factors, patient characteristics, tumor characteristics, previous cancer-related treatment factors and the overall top 9 were .90, .74, .87, .67 and .87, respectively, indicating moderate to strong agreement (Table 5). The three highest ranking factors were ‘epidural spinal cord compression (ESCC)’, ‘aggressive tumor behavior’ and ‘mechanical instability’ (Table 6).

**Table 5.** Ranked Risk Factors Identified in Round 3.

Rank	Risk Factor	Mean Ranking Score	Top-Half Rank, %	Kendall’s W
	**Radiological factors**			.90
1	Epidural spinal cord compression (ESCC)	1.08	98.7	
2	Mechanical instability	2.07	97.0	
3	Deformity (kyphosis, malalignment, etc)	3.17	90.4	
4	Vertebral body collapse	3.91	8.3	
5	Lytic lesion	4.93	3.3	
6	Midthoracic location	5.84	2.3	
	Patient characteristics*			.74
1	ASA classification ≥3	1.72	86.9	
2	Missing alarm symptoms/red flags	2.04	95.3	
3	Elderly patients	3.36	79.1	
4	Frailty	3.90	13.8	
5	Underestimating the urgency of symptoms	4.36	20.5	
6	Immunosuppression	5.83	3.7	
7	BMI <18.5 or >30.0	6.80	0.7	
	Tumor characteristics*			.87
1	Aggressive tumor behavior	1.06	95.6	
2	Involvement of ≥2 columns	2.01	3.4	
3	Osteodestructive tumor	2.93	1.0	
	**Previous cancer-related treatment factors**			.67
1	Absence of multidisciplinary team	1.66	86.3	
2	Persisting mechanical instability post-surgery	2.07	94.2	
3	Poor response to systemic treatment	2.87	94.5	
4	Deviation from treatment plan	4.18	4.8	
5	Exhausted radiotherapy options	4.74	8.5	
6	Refractory to prior surgery	5.47	11.6	

*If a list contains an uneven number of factors, the half with fewer factors is considered the top-half.

Abbreviations: ASA, American Society of Anesthesiologists; BMI, Body Mass Index.

**Table 6.** Top 9 Ranking of all Risk Factors Identified in Round 3.

Rank	Risk Factor	Mean Ranking Score	Top-Half Rank, %	Kendall’s W
	Top 9*			.87
1	Epidural spinal cord compression (ESCC)	1.09	99.0	
2	Aggressive tumor behavior	2.20	97.3	
3	Mechanical instability	3.01	99.0	
4	Deformity (kyphosis, malalignment, etc)	4.35	85.3	
5	Involvement of ≥2 columns	5.19	4.4	
6	Missing alarm symptoms/red flags	5.99	5.5	
7	Tumor compromising vascular supply spinal cord	6.92	3.4	
8	Osteodestructive tumor	7.66	4.1	
9	Vertebral body collapse	8.59	2.0	

*Because the list contains an uneven number of factors, the half with fewer factors is considered the top-half.

### Final List of Risk Factors

A final list of 17 factors was established after discussion of the Delphi study results within the expert panel, and definitions and classifications were established. These factors were divided into two categories: risk factors unanimously accepted by experts and supported by literature (Table 7) and risk factors suggested by experts, but currently not supported by literature (Table 8).

**Table 7.** Final List of Risk Factors Unanimously Accepted by Experts and Supported by Literature, With Definitions and Proposed Classification Method.

Factor	Definition	Proposed Classification Method
Epidural spinal cord compression (ESCC)	Metastatic process compressing spinal cord, cauda equina or nerve roots.^13^	Bilsky/ESCC scale^14^
Mechanical instability	Loss of spinal integrity as a result of a neoplastic process that is associated with movement-related pain, symptomatic or progressive deformity, and/or neural compromise under physiologic loads.^15^	SINS score^15^
Vertebral body collapse	Collapse of a vertebral body.^15^	Degree of vertebral body collapse, based on the SINS score (>50% collapse, <50% collapse, no collapse with >50% body involved, none of these)^15^
Lytic lesion	The type of metastatic lesion, ie, lytic, mixed or blastic lesion.	Type of bone lesion, based on SINS score (lytic, mixed, blastic)^15^
Midthoracic location	Location of the lesions on the spine between T3 to T10.	Metastatic lesion between T3 to T10 based on (semi-rigid) spine location in the SINS score - Yes/No^15^
ASA classification ≥3	Fitness for treatment.^16^	ASA classification^16^
Missing alarm symptoms/red flags	Red flags indicative of metastatic spinal disease in patients with a known history of cancer: new onset of back pain, nocturnal back pain, radicular back pain, mechanical pain.^17^	Red flag(s) missed -Yes/No^17^
Frailty	Age-associated decline in physiological reserve and reduced resilience to stressors resulting in adverse health outcomes.^18^	Modified frailty index,^19,20^ sarcopenia (TPA/VB)^21^
Involvement of ≥2 columns	Metastatic spinal disease in two or more columns.	Denis classification^22^

Abbreviations: ASA, American Society of Anesthesiologists; SINS, Spinal Instability Neoplastic Score; TPA/VB, total psoas area/vertebral body.

**Table 8.** Final List of Risk Factors Suggested by Experts, Currently Not Supported by Literature, With a Description and Suggested Classification Method.

Factor	Description	Suggested Classification Method
Deformity (kyphosis, malalignment, etc)	Metastatic spinal deformity includes scoliosis, hyperkyphosis and hypolordosis, each of which can lead to imbalance of the structural support of the spinal column.	A global and focal deformity measure (SVA/CVA, Cobb angle)^23^
Health status	A composite factor including but not limited to BMI <18.5 or >30.0, low albumin and karnofsky performance scale.	To be defined
Aggressive tumor behavior	Rapidly growing epidural tumor or an epidural tumor not responsive to current therapy. However, a patient is not at increased risk if there is a targetable mutation and therapy left.	To be defined
Persisting mechanical instability post-treatment	Remaining loss of spinal integrity as a result of a neoplastic process that is associated with movement-related pain, symptomatic or progressive deformity, and/or neural compromise under physiologic loads post-treatment.^15^	SINS score^15^
Implant failure	Presence of implant failure, for example screw pull out, rod breakage and cage subsidence.	Yes/No
Adherence to treatment plan made by a multidisciplinary team	Adherence to a well-described plan with input from the multidisciplinary team.	Yes/No
Tumor comprising vascular supply spinal cord	Number of segmental vessels involved (consecutive, spinal level and/or bilateral).	To be defined
Poor bone quality	Bone quality appearance on radiologic imaging.	Osteopenic or osteoporotic appearance on radiologic imaging

Abbreviations: BMI, Body Mass Index; CVA, coronal vertical axis; SINS, Spinal Instability Neoplastic Score; SVA, sagittal vertical axis.

## Discussion

This international Delphi study identified risk factors associated with the development and/or progression of neurological deficits in patients with metastatic spinal disease. Twelve radiological factors, fourteen patient characteristics, six tumor characteristics and twelve previous cancer-related treatment factors were identified. After ranking these factors in the consecutive rounds ‘epidural spinal cord compression (ESCC)’, ‘aggressive tumor behavior’ and ‘mechanical instability’ were considered the most important factors. This study provides valuable insights into the factors that may be predictive of the development of neurological deficits. The results will be of interest to clinicians and researchers as they will support prognostication and propose future directions for prognostication research in patients with metastatic spinal disease.

The three highest ranking factors from the current study can also be found in the NOMS framework.^24^ The NOMS framework is increasingly used by physicians in the field of spinal oncology to optimize decision-making and patient care in patients with metastatic spinal disease. NOMS consists of four considerations: neurologic, oncologic, mechanical and systemic assessment.^24^ ‘Epidural spinal cord compression (ESCC)’ was considered the most important risk factor for the development and/or progression of neurological deficits by the participants of the Delphi study. ESCC is also one of the three pillars that make up neurologic assessment in the NOMS, together with myelopathy and functional radiculopathy. In addition, ‘aggressive tumor behavior’ can be found in the oncological assessment, where the currently available line(s) of treatment and the expected response are considered. Lastly, for the mechanical component of NOMS, mechanical instability and whether stabilizing surgery is indicated is evaluated.^25^ Within NOMS, mechanical instability is classified by the SINS score.^15,26^

Interestingly, the radiological factor ‘tumor compromising vascular supply spinal cord’ was included in the overall top 9 ranking, but did not reach the top-half of the radiological factors ranking. An explanation might be that this factor could have fit within multiple categories. For example, other radiological factors than a tumor compromising the vascular supply may have been of more interest when considering individual radiological factors, but a tumor compromising the vascular supply may be of great importance when assessing the overall risk of neurological deficits.

In the current study, the potential interactions between factors listed by the participants in round 2 where numerous (806 interactions were proposed). In a future study, these interactions will be explored further, which may subsequently lead to new hypotheses regarding the etiology of neurological deficits in metastatic spinal disease.

This Delphi study has several strengths and limitations. Strengths of this study were the credibility and number of participants which were all active members of AO Spine the globally leading academic community for spine surgeons, inclusion of physicians from countries all over the world, and the use of the well-known Delphi approach. This has led to the generation of a broad and widely supported list of potential risk factors resulting from a transparent process.

A limitation of this study is the possibility of responder bias, which is present in all survey-based studies. Physicians specialized or with a special interest in metastatic spinal disease, could be more likely to respond to the survey. Furthermore, as these individuals are likely to be aware of the current paradigms in metastatic spinal disease, this may have influenced their response. Finding the three highest rankings factors from the current study also in the 17-year old NOMS framework could be an example of this type of bias. Additionally, the survey was distributed by AO Spine, reaching mostly orthopedic surgeons and neurosurgeons; however, an expert radiation oncologist in spine was prominent in the web conference discussions. It can be hypothesized that a larger variety of risk factors might be produced if respondents comprised of a more diverse group of physicians including more medical oncologists, radiation oncologists, radiologists and neurologists. Furthermore, geographical location could be of influence on the presentation and development of neurological deficits in patients with metastatic spinal disease. Despite distributing the questionnaires globally to all active AO Spine members, the vast majority of respondents originated from Europe and Asia, which could impact the generalizability of the risk factors.

Since the surveys were completed anonymously, it was not possible to invite back participants who had participated in previous rounds. In round 2, 53% of the participants did not participate in round 1, and 44% of the participants in round 3 did not participate before in the study. As described, response bias is common in survey studies. It may be that a selective group of participants may be more likely to respond in all rounds. Also, with anonymous responses, it was not possible to provide participants with their own response from the previous round. However, collated group responses from the previous round were provided to the participants so participants were able to compare their own opinion to group responses.

Lastly, the definition of factors may have played a role in the ranking and is a significant limitation in this study. As the participants of the Delphi study provided factors without giving a definition or further explanation, we were not able to further investigate the participant’s definition of the identified factors. For example, the factor ‘elderly patients’ was put forward often in the Delphi study, but most participants did not indicate at what age a patient is considered elderly. To further study these types of factors and ultimately use them in the clinical setting, clear definitions are necessary. Therefore, definitions of the highest-ranking factors and their classifications were proposed by an expert panel.

## Conclusion

Timely identification of patients with metastatic spinal disease who are at increased risk of developing neurological deficits can facilitate prevention of development and/or progression of neurological deficits.^2,4^ However, evidence on risk factors for the development and/or progression of neurological deficits is scarce and hence an evidence base for physicians, researchers and policy makers is lacking. This Delphi study provides a comprehensive and widely supported list of potential factors to more effectively study prognostication in metastatic spinal disease. The highest-ranked factors and interactions need validation from large datasets and may subsequently be used in the development of predictive models, to inform future randomized trials, as covariates in prognostic studies and ultimately to guide clinical practice.
